# Quality characteristics and antioxidant activity of the Korean traditional rice-oat wine “*Makgeolli*” supplemented with green seaweed *Codiumfragile*

**DOI:** 10.1016/j.heliyon.2024.e39348

**Published:** 2024-10-12

**Authors:** Eun Hee Park, Eun Bi Jeon, Pantu Kumar Roy, Sung-Hee Park, Shin Young Park

**Affiliations:** a Department of Seafood Science and Technology, Institute of Marine Industry, Gyeongsang National University, Tongyeong, 53064, Republic of Korea; b Practical Technology Research Group, World Institute of Kimchi, Gwangju, 61755, Republic of Korea

**Keywords:** Rice-oat *Makgeolli*, *Codium fragile*, Fermentation, Physicochemical property, Microbiological property, Antioxidant activity

## Abstract

In this study, we prepared *Makgeolli*, a traditional Korean alcoholic beverage, by adding 20 % raw *Codium fragile* (Cf) to a base of rice and oats (1:1 ratio) (COM). We analyzed its quality characteristics and antioxidant activity. The rice-based *Makgeolli* with Cf was designated as the control (CRM). We assessed the physicochemical (pH, acidity), microbiological (yeast, lactic acid bacteria), antioxidant (DPPH, ABTS), and taste properties (7-point hedonic scale) of CRM and COM during a 7-day fermentation. The pH/acidity levels of CRM and COM reached 3.66/0.68 and 3.69/0.95, respectively, after 7 days of fermentation. Compared with CRM, COM had a lower alcohol and sugar content (*P* < 0.05) (CRM: 14.5 %/9.8 Brix, COM: 11.2 %/7.4 Brix). However, COM contained more yeasts and lactic acid bacteria (*P* < 0.05) (CRM:4.30/4.28 log, COM: 6.04/6.09 log) than CRM. The antioxidant activity of DPPH/ABTS (*P* < 0.05) was approximately 1.4 times higher in COM than in CRM (CRM: 40.00/51.57 %, COM: 56.76/70.91 %). In addition, COM had an excellent taste preference score (*P* < 0.05) due to its overall fresh flavor and taste (CRM: 4.93, COM: 6.06). Therefore, the results demonstrated that COM is a *Makgeolli* with enhanced antioxidant function against yeast and lactic acid bacteria, as well as improved flavor, suggesting the possibility for commercialization.

## Graphical abstract

**Figure:**
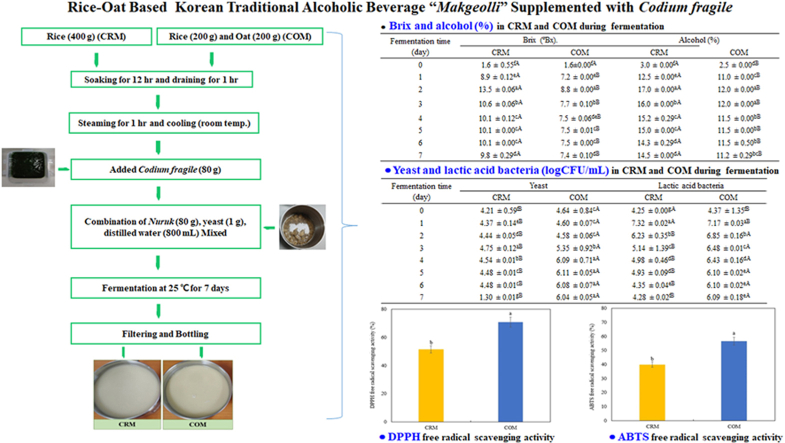

## 1 Introduction

*Makgeolli* is representative traditional liquor in Korea. It is also called *Takju* or Nongju, as it is mainly consumed by farmers while doing farming work [1]. *Makgeolli* is a traditional fermented food recognized for its nutritional value as a fermented liquor made through the parallel double-fermentation of saccharification and fermentation by adding *Nuruk* (a traditional Korean fermentation starter) and water to grains such as glutinous rice and barley [2]. *Nuruk* which consists of wheat and rice is a fermentation starter culture used to make alcoholic beverages from grains and allows various natural microorganisms such as mold, yeast, and lactic acid bacteria (LAB) to grow [3]. *Nuruk* is a traditional starter culture made from wheat, rice, or grits, which allows for the growth of various natural types of microorganisms such as fungi, yeast, and lactic acid bacteria, which are useful in the saccharification of the rice starch during fermentation. *Aspergillus kawachii*, *Aspergillus oryzae*, and *Rhizopus* sp., among other raw materials, directly or indirectly transform the majority of the volatile compounds produced during a *Makgeolli* fermentation using *Nuruk* yeast into organoleptic characteristics. It is classified as improved yeast made by pure culturing and inoculating bacteria such as yeast, and its enzyme activity, organic acid production ability, and alcohol fermentation ability vary depending on the type of yeast and the ratio of the mixture [3,4].

The flavor of fermented *Makgeolli* is such that the five tastes of sweet, sour, spicy, bitter, and astringent are in harmony [5]. It is a national alcoholic beverage that continues to receive public attention and devotion. Commercial rice wine (6–8% alcohol) has a low alcohol content, which is not burdensome to the body, and when consumed in moderate amounts, it promotes blood circulation and contains various nutritional ingredients such as yeast and lactic acid bacteria. In addition, *Makgeolli* is known to contain the essential amino acids lysine, leucine, and arginine, as well as acetylcholine, which support liver function [6]. In recent years, the perception of drinking has changed, with healthier and more “light-hearted” drinking becoming increasingly popular. People generally avoid beverages that contain a high percentage of alcohol (an alcohol content of 16.9 % or higher, such as soju) in favor of low-alcoholic beverages and non-alcoholic beverages. As a result, the steady growth in the market for traditional liquor has boosted consumer interest in unique beverages such as *Makgeolli,* and prompting active preliminary research [7]. With a low alcoholic content of around 6 %, *Makgeolli* is believed to have probiotic properties, containing 100–500 times more *Lactobacillus* bacteria than yogurt and exhibiting antioxidant, anti-cancer, anti-inflammatory, anti-aging and anti-colic properties by reducing bad cholesterol levels and increasing immune and circulatory systems [1,8].

*Codium fragile* (Cf) is a member of the green algae family, which is widely distributed in coastal areas around the world, including along the coasts of Korea, Japan, China, the Philippines, Hawaii, and Africa. Although Cf is mainly used as food, it is not as widely known as other seaweeds such as sea mustard, sea tangle, and laver [9]. In addition, it is often used as a supplementary ingredient in kimchi in the southern regions of Korea because of its distinctive sea flavor and odor neutralizing effect [1]. In the case of living organisms, most of the general components of Cf are water. Carbohydrates are next at 2.4 %, and in the case of the dried product, carbohydrates are the highest at 52.8 %, followed by ash at 22.6 %. In addition, the inorganic salt content is very high at 40 mg/100 g (dry weight) of calcium, and in terms of vitamins, the content of vitamin C is high at 9 mg/100 g (dry weight). In addition, the insoluble portion accounts for most of the dietary fiber at 29.4 %, and Cf contains various amino acids [10] Cf extracts can be used not only as prebiotic materials but also as bioactive substances for health promotion purposes by increasing their antioxidant activity [11]. Researchers have also been reported that Cf extracts contain polyphenol and flavonoid compounds, which serve as functional materials due to their antioxidant properties [12] and the presence of various bioactive substances [13]**.** Cf extracts were concluded to possess strong antioxidant power by inhibiting the synthesis of nitric oxide because of the large number of phenolic compounds they contain [8]. Acrylic acid in the extract has a unique antibacterial effect that suppresses only harmful bacteria and is rich in beta-carotene, which suppresses cancer cells, increases immunity, and contains anticoagulant active substances, as well as being a strong antibacterial agent against bacteria. It is a useful seaweed that can be applied in various fields as it has been found to have antipyretic, detoxification, and immune activity properties [14].

Oats (*Avena sativa* L.) is a winter crop that belongs to the rice family and is rich in protein and fat compared with other grains. In addition, about 70–80 % of the protein is globulin, which has a high content of essential amino acids such as lysine [15]. Furthermore, studies have reported the anti-inflammatory and anti-cancer effects of avenanthramide, found exclusively in oats. Researchers have proven its anti-inflammatory effect by suppressing NO production and reducing the expression of iNOS, an inflammatory factor [16].

Researchers have also confirmed the excellent antioxidant properties of the polyphenols found in oats [17]. In addition, unsaturated fatty acids and beta-glucan in dietary fiber are also present in large amounts, and the University of Moscow reported that the consumption of oatmeal has the ability to detoxify the body of heavy metals [18]. Oats, listed among the world's top 10 health foods in 2002 and recognized as the only superfood among grains in 2009, have garnered recognition for their outstanding nutritional and health-functional values. Notably, oats are now integral to low-calorie diets, reflecting evolving consumption preferences that prioritize grains rich in dietary fiber over simple sugars. Consequently, the surge in research initiatives and the introduction of high-functional processed products centered around oats have led to a noteworthy increase in both the consumption and production of this grain [19].

Since 2008, *Makgeolli's* market size has grown globally due to the well-being trend and the spread of K-POP culture [20]. Researchers are conducting preliminary studies to investigate the production of *Makgeolli* with the addition of fruit and vegetables such as pears [21], cucumbers [22], blueberries [23], lotus leaves [24], quinoa [25], oats [26], and black rice [27]. Recent research related on fermented beverages have examined kombucha [28], plant-based fermented beverages [29], fermented mare's milk [30], and oat beer, which uses oats to produce fermented products [31]. However, although research has been conducted to identify the characteristics of these compositions, no case has yet been reported in which *Makgeolli* was manufactured by mixing seaweed and oats.

Therefore, in our study, we explored the development of a new strategy for *Makgeolli* that indicates a Cf supplement. The physicochemical (pH, acidity, alcohol, and sugar content) and microbiological (yeast and lactic acid bacterial count) properties as well as the antioxidant activity (DPPH/ABTS) were analyzed by fermenting the rice-oat-based Korean wine *Makgeoll*. Through comprehensive analysis of physicochemical and microbiological properties, as well as antioxidant activity, this research aims to provide valuable insights into the development of a health-enhancing and uniquely flavored *Makgeolli*.

## 2 Materials and methods

### 2.1 Raw materials

The Cf used in this experiment was collected from an oyster farm in Changho-ri, Sadeong-myeon, Geoje-si, Gyeongsangnam-do (N 34° 54.4068′, E128° 29.2503′). The main ingredients of *Makgeolli* were purchased from a local market in Hadong-gun, Gyeongsangnam-do (rice), and from Hyundai Nongsusan in Pocheon-si, Gyeonggi-do, Korea (oats). The *Nuruk* (Korean-style bran *Koji*), made from Korean wheat, was purchased from Songhaggogja (Gwangju, Korea), and the yeast was purchased from Songcheon Yeast (Songcheon fermentation, Cheongyang-gun, Chungcheongnam-do, Korea). *Nuruk* contains half and half of the *Aspergillus luchuensis* and *Aspergillus oryzae* chrysanthemum bacteria. According to Korea's national intangible cultural asset on June 15, 2021, *Makgeolli* was prepared for this study [14].

### 2.2 Production of rice-oat Makgeolli with Cf

**Fig. 1:**
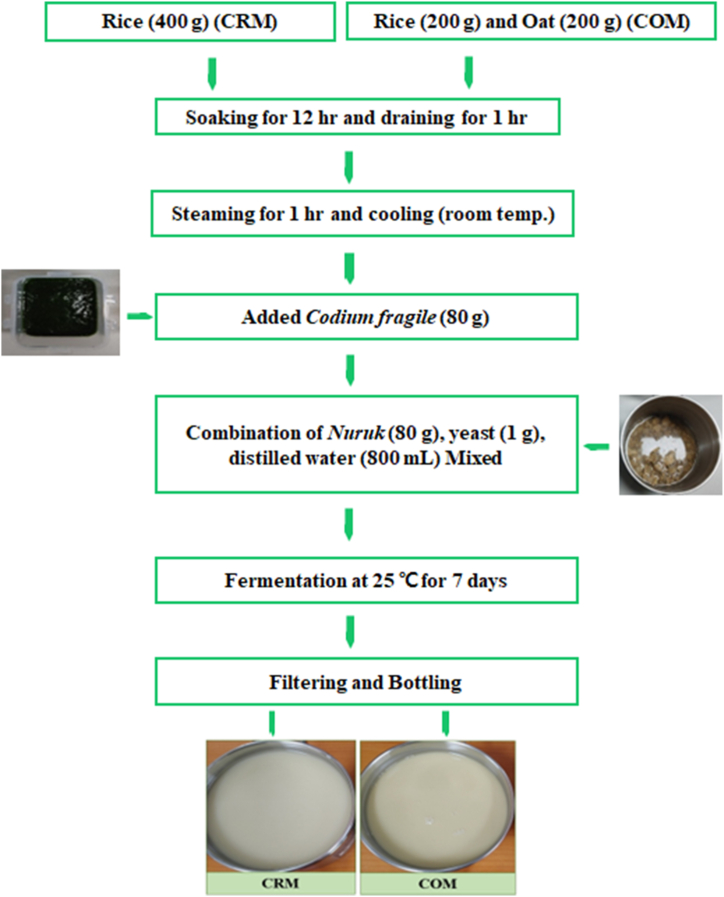
A flow diagram for the preparation of *Makgeolli* with *Codium fragile* + Oat. CRM: *Codium fragile +* Rice-based *Makgeolli;* COM: *Codium fragile* + Oat-based *Makgeolli*.

The manufacturing process of rice-oat *Makgeolli* is schematically shown in Fig. 1. Rice-oat *Makgeolli* was prepared by washing the rice and oats until the water remained clear. After soaking the rice and oats in water for approximately 12 h, we placed them on a sieve for an hour to allow the water to drain. The soaked rice and oats were placed in a cotton cloth, placed in a steamer, steamed at 100 °C for 1 h, and then allowed to cool to room temperature before use. For the control group, *Makgeolli* (CRM) to which Cf had been added was prepared as follows: 400 g of cooked rice and 100 mL of lukewarm water inoculated with 80 g of *Nuruk* at 40 °C the day before, 1 g of yeast, 80 g of Cf, and 600 mL of water were homogenized in a sterilized glass bottle and fermented at 25 ± 1 °C for 7 days. In a previous study [14], the result of DPPH/ABTS radical scavenging ability was the highest in the Cf-added group of 20 % (80 g). For the experimental group, rice and oat *Makgeolli* (COM) supplemented with Cf was prepared as follows: 400 g of cooked rice and oats (1:1), 100 mL of lukewarm water inoculated with 80 g of *Nuruk*, at 40 °C the day before, 1 g of yeast, 80 g of Cf, and 600 mL of water were homogenized in a sterilized glass bottle and fermented at 25 ± 1 °C with stirring once a day for 2 days. The Cf used in this study was prepared and added after grinding in a blender with 100 mL of water, and the total amount of water used for making *Makgeolli* was 800 mL. The top of the bottle was covered with plastic wrap and a rubber band, placed in an incubator set at 25 °C, and allowed to ferment for 7 days. The samples were filtered (SUPRA22K, Hanil Science Industrial Co., Seoul, Korea) before testing. The physicochemical properties, microbiological properties, antioxidant activity, and taste tests of *Makgeolli* in the control (CRM) and experimental (COM) groups were investigated.

### 2.3 Analysis of physicochemical quality characteristics

#### 2.3.1 pH value and acidity

A *Makgeolli* sample (15 mL) was centrifuged (SUPRA22K, Hanil Science Industrial Co., Korea), and the pH value of the supernatant was measured using a pH meter (A211, Thermo Orion, Benchtop, MI, USA). We determined the acidity by diluting 10 mL of the sample 10-fold. Next, we collected 20 mL of the diluent in a 50 mL Erlenmeyer flask, added 2–3 drops of 1 % phenolphthalein indicator, and titrated the solution with 0.1 N NaOH until it turned pink. We calculated the acidity as the lactic acid content (%) using the following equation [14].

Acidity(%)=(0.009×NaOHtitrationvolume(mL)×NaOHtiter×dilutionfactor)/(samplevolume(mL))×100

#### 2.3.2 Brix and alcohol content

We determined the Brix and alcohol content by analyzing the supernatant, which we obtained by centrifuging the sample with a centrifuge (SUPRA22K, Hanil Science Industrial Co., Korea), using an electronic saccharimeter (PAL-1, ATAGO CO., Japan) during fermentation, and expressing the results as Brix. The alcohol content was measured according to the National Tax Service's Liquor Analysis Regulations (2010). We achieved this by quantifying 100 mL of the sample in a mass cylinder, then transferring it to a distillation flask and heating it. The flask was then connected to a condenser and the liquid was distilled until the volume of the distillate reached 70 mL. We discontinued the distillation at this point and supplemented the distillate with 30 mL of distilled water. We measured the alcohol content using an alcoholmeter (211-DK-12; Daekwang, Korea). The alcohol content was expressed as a percentage (v/v) [14].

### 2.4 Analysis of microbiological quality characteristics

The yeast count and number of lactic acid bacteria were determined by diluting 1 mL of the sample in sterile physiological saline according to the decimal dilution method. Yeast counts were determined by mixing 1 mL of the diluted sample and potato dextrose agar (Difco Co., Detroit, MI, USA) evenly in a Petri dish, followed by incubation at 25 ± 1 °C for 5−7 days before counting. The lactic acid bacteria were counted by mixing 1 mL of the diluted sample and lactobacilli MRS (Difco Co., Detroit, MI, USA) thoroughly in Petri dishes and counting the yellow colonies after 24−48 h of incubation at 37 °C. The colonies that were produced were expressed as colony-forming units (CFU/Ml) [**25**].

### 2.5 Analysis of antioxidant quality characteristics

#### 2.5.1 DPPH radical scavenging ability

The DPPH [1-diphenyl-2-picrylhydrazyl] (Sigma-Aldrich Co., St. Louis, MO, USA) radical scavenging activity was measured using the method of Kang et al. (2016) [32]. A solution was prepared for measurement by adding 800 μL of a 1.5 × 10^−4^ M DPPH solution to 200 μL of the centrifuged fermentation supernatant (diluted fourfold), vortexed for 20 s, and stored in a refrigerator for 30 min. The absorbance was measured at 517 nm using a spectrophotometer (Spectronic2D, Thermo Electron Co., Waltham, MA, USA). The DPPH radical scavenging activity was expressed as a percentage (%) as shown below by calculating the absorbance of the CRM and COM [14].

$$\text{DPPH}\;\text{radical}\;\text{scavenging}\;\text{activity}\;\left(\%\right)=\left(A_{517}\;\text{of}\;\text{control}-A_{517}\;\text{of}\;\text{sample}\right)/\left(A_{517}\;\text{of}\;\text{control}\right)\times 100$$

#### 2.5.2 ABTS radical scavenging ability

A solution was prepared by dissolving 7 mM and 2.45 mM of potassium persulfate in distilled water. This solution was left to stand in the dark for 14−16 h, during which time the ATBS cation radical (ATBS^+^) [2′-Azino-bis diammonium (3-ethylbenzthiazoline-6-sulfonic acid)] (Sigma-Aldrich Co., St. Louis, MO, USA) was generated. The solution was diluted with ethanol, and the absorbance was adjusted to 0.750 ± 0.002 at 415 nm by mixing with anhydrous ethanol in a 1:1 ratio. After adding 3 mL of COM to 1 mL of the diluted ATBS^+^ solution and allowed to react for 20 min, after which the absorbance at 415 nm was measured using a spectrophotometer (Spectronic2D, Thermo Electron Co., Waltham, MA, USA) [14].

$$\text{ATBS}\;\text{radical}\;\text{scavenging}\;\text{activity}\;\left(\%\right)=\left(A_{415}\;\text{of}\;\text{control}-A_{415}\;\text{of}\;\text{sample}\right)/\left(A_{415}\;\text{of}\;\text{control}\right)\times 100$$

### 2.6 Taste tests

A total of 16 adults (8 men and 8 women) were selected as panelists for the taste tests, including undergraduate and graduate students of the Department of Seafood Science and Technology at Gyeongsang National University. *Makgeolli* with an alcohol content of 7∼8 % was prepared using 2 parts water to 1 part filtered stock solution fermented for 7 days. After storing the *Makgeolli* at 4 °C for 24 h, each panelist was provided with about 20 mL in a paper cup, and water for rinsing their mouths after tasting each sample. The results of the taste tests of *Makgeolli* were expressed by surveying the color, flavor, taste, appearance, and overall acceptability. Each item was evaluated on a 7-point scale (7 points for very good, 1 point for very bad).

### 2.7 Statistical analysis

All analyses were performed in triplicate, and the data were expressed as mean ± SD (standard deviation). We performed ANOVA and Duncan's multiple range tests using SPSS version 12.7 software (SPSS Inc., Chicago, IL, USA). Duncan's multiple range test was used to compare the differences among mean values. One-way ANOVA was performed to evaluate the statistical significance of the differences between the CRM and COM samples. The statistically significant difference at probability level of 5 % (*P* < 0.05).

## 3 Results and discussion

### 3.1 Changes in pH and total acid content during fermentation

The acidity and pH measurements that were made at daily intervals for 7 days while *Makgeolli* (CRM, COM) was undergoing fermentation are provided in Table 1. Acidic substances produced during the fermentation period affect the pH of *Makgeolli* [33]. This factor indicates changes in the composition of *Makgeolli* and is an important indicator of the fermentation process, which is a complex process that involves the production of alcohol [34]. The pH of COM was 4.85 on day 0 of fermentation and was significantly lower at 3.63 on day 2 of fermentation (*P* < 0.05). After this, the pH stabilized without any major changes until the final 7 day. Although the pH of CRM was 5.28 on day 0 of the fermentation process, which was higher than that of COM, the pH of CRM changed according to the same pattern as that observed for COM (Table 1). That is, the pH of both CRM and COM decreased rapidly on day 2 of fermentation. This result was consistent with that of Kim et al. [35] and Park et al. [36] reported that the pH decreased on day 2 of fermentation during the production of *Makgeolli*. The change in pH during *Makgeolli* fermentation is greatly affected by the total acid content, and carbon dioxide produced as a by-product of alcohol fermentation is discharged into the atmosphere and does not have a significant effect on pH [37]. In general, during the fermentation process of makgeolli, organic acids and alcohol are produced by microorganisms, causing the pH to rapidly decrease. This is thought to be related to the rapid increase in the alcohol content produced after 2 days of fermentation, leading to good yeast growth, and is due to various organic acids produced during fermentation. Additionally, the pH of 3.66 for CRM and 3.69 for COM on day 7 of fermentation was similar to the pH range of 3.40–3.77 of commercially available unpasteurized *Makgeolli*. Kim et al. [27] noted that the pH of black rice *Makgeolli* fermented for 10 days was 4.03–4.23. The pH results of this study were within the standard range of 3.8–4.7, which is the pH range of rice wine according to the Liquor Tax Act.

**Table 1:** Changes in pH and acidity (%) in CRM and COM during fermentation.

Fermentation time (day)	pH		Acidity (%)	
	CRM	COM	CRM	COM
0	5.28 ± 0.00^aA^	4.85 ± 0.00^aB^	0.20 ± 0.00^eA^	0.19 ± 0.00^eB^
1	5.08 ± 0.01^bA^	4.69 ± 0.00^bB^	0.27 ± 0.03^fA^	0.19 ± 0.05^eB^
2	3.57 ± 0.01^fB^	3.63 ± 0.01^efA^	0.86 ± 0.01^aA^	0.82 ± 0.02^aB^
3	3.54 ± 0.02^gB^	3.62 ± 0.01^fA^	0.86 ± 0.01^aA^	0.82 ± 0.02^aB^
4	3.58 ± 0.01^fB^	3.64 ± 0.01^eA^	0.80 ± 0.02^abA^	0.77 ± 0.01^bB^
5	3.62 ± 0.02^eB^	3.66 ± 0.00^dA^	0.78 ± 0.01^bcA^	0.71 ± 0.01^cB^
6	3.69 ± 0.01^cB^	3.67 ± 0.01^dA^	0.72 ± 0.00^cdA^	0.67 ± 0.02^dB^
7	3.66 ± 0.03^dB^	3.69 ± 0.01^cA^	0.68 ± 0.02^dA^	0.65 ± 0.01^dB^

The data indicates means with standard deviations (three samples/treatment). Within the same column, means with different letters (a-f for each fermentation time). Within the same row, means with different letters (A-B). The one-way ANOVA performed by *t*-test was carried out to evaluate the statistical significance of differences between CRM and COM samples.

CRM: *Codium fragile* + Rice-based *Makgeolli*; COM: *Codium fragile* + Rice/Oat-based *Makgeolli.*

The acidity, which affects the pH of *Makgeolli*, is directly related to its taste and flavor of *Makgeolli* and can also affect its preservation [38]. On day 0 of the fermentation, the acidity of CRM and COM was 0.20 % and 0.19 %, respectively. Like the variation in the pH of CRM and COM, the acidity (CRM: 0.86, COM: 0.82) increased rapidly on the second day of fermentation. In a study on purple sweet potato rice malt *Makgeolli*, as fermentation progressed, the acidity increased because of the production of organic acids such as malic acid, succinic acid, and citric acid by the action of yeast and lactic acid bacteria [39]. The high acidity level on the day 2 of fermentation is due to the addition of water to the yeast the day before to speed up the initial fermentation to activate the bacteria in the yeast. After two days of fermentation, the acidity gradually decreased to 0.86, 0.80, 0.78, 0.72, and 0.68 % for CRM and 0.82, 0.77, 0.71, 0.67, and 0.65 % for COM from the third to the seventh day of the final fermentation. This mirrored the results of a study in which changes in the quality were observed during the fermentation of oat *Makgeolli* made with different amounts of water [26]. Meanwhile, a comparison of the change in the pH and acidity of COM and CRM revealed an ideal negative correlation, with increasing acidity and decreasing pH.

### 3.2 Changes in the sugar content during fermentation

The measurements of the sugar content of CRM and COM are presented in Table 2. On day 0 of the fermentation, the sugar content of CRM and COM was the same at 1.6 Brix. On day 1 of fermentation, this value increased rapidly to 8.5 and 7.2 for CRM and COM, respectively. The rapid increase in sugar content on the 1 day of fermentation is attributed to the method that was initially used to accelerate the fermentation by adding malt and yeast to water a day before soaking the starch in grains, which are ingredients of *Makgeolli*, to activate the bacteria in the yeast. On day 2 of fermentation, the Brix values of CRM and COM were 13.5 and 8.8, respectively. According to data from the National Institute of Agricultural Sciences, the carbohydrate content per 100 g is 78.74 g for rice and 66.66 g for oats. Therefore, COM has a lower sugar content than CRM because rice contains more carbohydrates, which determine the sugar content [40]. Meanwhile, after the initial rapid increase in the sugar content, it gradually decreased to 10.6, 10.1, and 9.8 Brix for CRM and 7.7, 7.5, and 7.4 Brix for COM from the third to the final day (7 day) of fermentation. This is the result of brown rice produced under different fermentation conditions. Another study on *Makgeolli* [41] also found that the sugar content increased on the first and second days of fermentation, after which it gradually decreased, which was consistent with the results of this study. The representative sweetness of *Makgeolli* are maltose and glucose. After the yeast consumes the sugar, the Brix content decreases and the alcohol content increases. The significant decrease in glucose content reflects conversion to ethanol by yeast [25]. The tendency for the sugar content to decrease is the result of the decomposition of starch into sugar due to the action of saccharification amylase and the nutrient source of yeast or fermentation. As fermentation progresses with the substrate for a certain period, the sugar content decreases, and the alcohol content increases [2].

**Table 2:** Changes in sugar content (Brix) and alcohol (%) in CRM and COM during fermentation.

Fermentation time (day)	Sugar content (°Brix)		Alcohol (%)	
	CRM	COM	CRM	COM
0	1.6 ± 0.55^fA^	1.6 ± 0.00^fA^	3.0 ± 0.00^fA^	2.5 ± 0.00^dB^
1	8.5 ± 0.12^eA^	7.2 ± 0.00^eB^	12.5 ± 0.00^eA^	11.0 ± 0.00^cB^
2	13.5 ± 0.06^aA^	8.8 ± 0.00^aB^	17.0 ± 0.00^aA^	12.0 ± 0.00^aB^
3	10.6 ± 0.06^bA^	7.7 ± 0.10^bB^	16.0 ± 0.00^bA^	12.0 ± 0.00^aB^
4	10.1 ± 0.12^cA^	7.5 ± 0.06^deB^	15.2 ± 0.29^cA^	11.5 ± 0.00^bB^
5	10.1 ± 0.00^cA^	7.5 ± 0.01^cB^	15.0 ± 0.00^cA^	11.5 ± 0.00^bB^
6	10.1 ± 0.00^cA^	7.5 ± 0.00^cB^	14.3 ± 0.29^dA^	11.5 ± 0.50^bB^
7	9.8 ± 0.29^dA^	7.4 ± 0.10^dB^	14.5 ± 0.00^dA^	11.2 ± 0.29^bcB^

The data indicates means with standard deviations (three samples/treatment). Within the same column, means with different letters (a-f for each fermentation time). Within the same row, means with different letters (A-B). The one-way ANOVA performed by *t*-test was carried out to evaluate the statistical significance of differences between CRM and COM samples. CRM: *Codium fragile* + Rice-based *Makgeolli*; COM: *Codium fragile* + Rice/Oat-based *Makgeolli.*

### 3.3 Changes in alcohol content during fermentation

The alcohol content of *Makgeolli* is one of the factors that greatly affects the quality of *Makgeolli* and is an important ingredient that affects its flavor and preservation [37]. The alcohol content is determined by the production of ethanol during the process in which yeast decomposes sugar saccharified by fermentation. As fermentation progresses, the alcohol content increases because of an increase in the ethanol content [42]. The variation in the alcohol content during the fermentation of COM is presented in Table 3. The production of alcohol started on day 0 of the fermentation at 3.0 % for CRM and 2.5 % for COM, whereupon it rapidly increased to 12.5−11.0 % on the first day of fermentation to reach the highest level (17.0−12.0 %) on the second day of fermentation. This is the result of a rapid increase in the sugar content and a reduction in sugar content on the days 1 and 2 of fermentation, resulting in the production of ethanol with the aid of yeast [43.] In general, unlike the cases of *Polygonatum odoratum* [42] and red beans [43], in which the alcohol content gradually increased as fermentation progressed beyond 2–3 days, in this study, it gradually decreased to reach 14.5 % for CRM and 11.2 % for COM after 7 days. Cho et al. reported that the amount of alcohol produced differed according to the saccharification power of yeast [**39**]. The results showed that the alcohol content of COM was lower at 14.5−11.2 % compared with that of CRM, but methods were introduced to increase the initial fermentation speed and *Cf*. The sugar content of COM was 7.5 Brix and the alcohol content was 11.5 % on days 4–6 of the fermentation, after which it decreased to 7.4 Brix and 11.2 % on day 7. According to Kim et al. reported, the point at which the sugar content no longer decreased signaled the end of fermentation [44]. In this study, the difference in the starch content of oats in the experimental group compared with the control group first depleted the sugar, a source of nutrients for the yeast, and the COM fermentation reached its end point after 4−6 days when the yeast was failed to ferment alcohol. The results obtained from the experimental group COM are believed to be the result of adding yeast and yeast to water a day before soaking *Makgeolli* and using it when soaking *Makgeolli*, which promoted fermentation.

**Table 3:** Changes of yeast (log CFU/mL) and lactic acid bacteria (log CFU/mL) in the CRM and COM during fermentation.

Fermentation time (day)	Yeast		Lactic acid bacteria	
	CRM	COM	CRM	COM
0	4.21 ± 0.59^gB^	4.64 ± 0.84^cA^	4.37 ± 0.00^eA^	4.20 ± 1.35^fB^
1	4.37 ± 0.14^eB^	4.60 ± 0.07^cA^	7.32 ± 0.02^aA^	7.17 ± 0.03^aB^
2	4.44 ± 0.05^dB^	4.58 ± 0.06^cA^	6.23 ± 0.35^bB^	6.85 ± 0.16^bA^
3	4.75 ± 0.12^aB^	5.35 ± 0.92^bA^	5.14 ± 1.39^cB^	6.48 ± 0.01^cA^
4	4.54 ± 0.01^bB^	6.09 ± 0.71^aA^	4.98 ± 0.46^dB^	6.43 ± 0.16^dA^
5	4.48 ± 0.01^cB^	6.11 ± 0.05^aA^	4.93 ± 0.09^dB^	6.10 ± 0.02^eA^
6	4.48 ± 0.01^cB^	6.08 ± 0.07^aA^	4.35 ± 0.04^eB^	6.10 ± 0.02^eA^
7	4.30 ± 0.01^fB^	6.04 ± 0.05^aA^	4.28 ± 0.02^fB^	6.09 ± 0.18^eA^

The data indicates means with standard deviations (three samples/treatment). Within the same column, means with different letters (a-f for each fermentation time). Within the same row, means with different letters (A-B). The one-way ANOVA performed by *t*-test was carried out to evaluate the statistical significance of differences between CRM and COM samples. CRM: *Codium fragile* + Rice-based *Makgeolli*; COM: *Codium fragile* + Rice/Oat-based *Makgeolli.*

### 3.4 Changes in the number of yeast and lactic acid bacteria during fermentation

The change in the yeast count during fermentation for 7 days is presented in Table 3. Immediately after soaking, the yeast count of CRM increased from 4.21 log_10_ CFU/mL on day 0 of fermentation to a maximum of 4.75 log_10_ CFU/mL on day 3 of fermentation, followed by a gradual decrease to 4.54−4.30 log_10_ CFU/mL on the 4–7 days of fermentation. Changes in yeast and lactic acid bacteria numbers during fermentation. Seo et al. [16] reported that general bacteria, molds, and yeast derived from yeast in the early stage of fermentation were in a symbiotic relationship, and the environment became acidic as lactic acid was produced due to the growth of lactic acid bacteria, resulting in an explosive increase in yeast.

In a study on changes in the yeast content during the fermentation period of *Makgeolli* [**5**], the yeast count increased rapidly from the third day, thereby following a trend similar to that in this study. However, according to Park et al. [45], the yeast count reached its maximum value on the fourth day of fermentation. In our study, COM had a maximum value of 6.11 log_10_ CFU/mL on the 5 days of fermentation; thus, COM reached its maximum value one day later. In addition, unlike CRM, which decreased to 4.30 log_10_ CFU/mL on the 7 days of fermentation, COM gradually decreased to 6.04 log_10_ CFU/mL on the final day of fermentation. This is consistent with the study on *Makgeolli* by Jeon et al. [**14**] in which the decrease in the yeast count was attributed to the inhibitory effect of alcohol produced by fermentation. In our study, the effect of increasing the initial fermentation rate by adding water to the yeast a day before soaking is thought to be responsible for promoting fermentation. A comparison between COM and CRM revealed that the former reached the maximum yeast value one day later than the latter, which is thought to be due to the differences in the supply of nutrient sources as well as the dependence of the fermentation ability on the type of grain used for making *Makgeolli* [42].The change in the lactic acid bacteria count during fermentation for 7 days is presented in Table 3. The difference between CRM (4.37 log_10_ CFU/mL) and COM (4.20 log_10_ CFU/mL) was insignificant in terms of the lactic acid bacteria counts on day 0 (*P* > 0.05). On day 1, the counts increased rapidly to 7.32 and 7.17 log_10_ CFU/mL for CRM and COM, respectively. On day 7, the final day of fermentation, the number of lactic acid bacteria in COM was about 1.5 times higher (*P* < 0.05) than in CRM (CRM; 4.28 log_10_ CFU/mL, COM: 6.09 log_10_ CFU/mL). According to food nutrition data from the National Institute of Agricultural Sciences, the total dietary fiber content per 100 g is 1.9 g for white rice and 18.8 g for oats [**40**]. The higher fiber content of oats is the reason for the higher bacterial count of COM than CRM, in that the dietary fiber becomes food for the lactic acid bacteria. In addition, Nile et al. [**2**] reported that *Makgeolli* provides a complex probiotic effect owing to the presence of lactic acid bacteria and yeast and lactic acid bacteria to proliferate because of their role as prebiotics, which constitute food for lactic acid bacteria, compared with the *Makgeolli* that is generally distributed in the market. Oat is known as a prebiotic with the ability to support the growth of microorganisms. Microorganisms tend to adhere to surfaces of prebiotic fibers. As a result, the environment of the carrier (e.g., oats) tends to provide a protective effect to the immobilized microorganisms. Microbes of prebiotics get a suitable environment for growth and viability during fermentation. Prebiotics inducing the growth or activity of beneficial bacteria probiotics producing short-chain fatty acids (SCFA) have lately received wide recognition for their beneficial influence on host intestinal microbiota and metabolic health. Some non-starch polysaccharides (NSP) are defined as prebiotics and oats being one of richest sources of NSP in grains are considered as potentially having prebiotic effect [46]. It is generally known that 700mLof commercially available *Makgeolli* contains more than 7 × 10^1^° lactic acid bacteria. *Makgeolli* is an alcoholic beverage in which various lactic acid bacteria and yeast are alive [47]. Although this study did not conduct experiments on the function of lactic acid bacteria as probiotics, Park et al. [48] reported that the lactic acid bacteria species isolated from commercial raw *Makgeolli* were strains suitable for the intestinal environment and met the selection criteria for probiotics.

### 3.5 Changes in the antioxidant activity (DPPH/ABTS radical scavenging activity) during fermentation

**Fig. 2:**
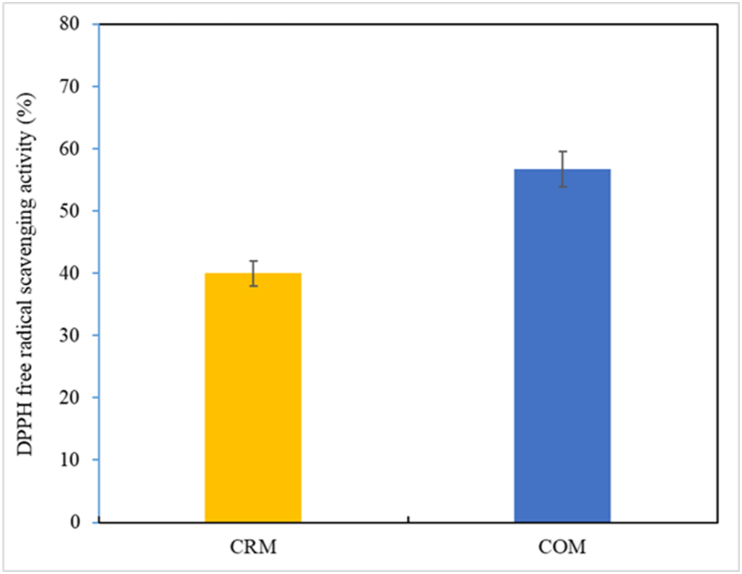
DPPH free radical scavenging activity in COM and CRM. The data indicates means with standard deviations (three samples/treatment). CRM: *Codium fragile* + Rice-based *Makgeolli;* COM: *Codium fragile* + Oat-based *Makgeolli*. ^a-b^At the same temperature and pH treatment, values marked with different superscript letters are significantly different *(P < 0.05).*

The results of measuring the DPPH radical scavenging activity of CRM and COM on day 7, the final day of fermentation, are shown in Fig. 2. The activity of COM was found to be approximately 1.5 times higher at 56.71 % than that of CRM at 40.00 %. This is consistent with a research report [**49**] that oats contain reductone, which stabilizes free radicals and terminates oxidation reactions in an antioxidant study of oat methanol extract. In a study [2] on *Makgeolli* prepared by adding 10–30 % of Cf, the DPPH activity of the group to which Cf had been added was about 2.4–2.7 times higher than that of the Cf -free group.

**Fig. 3:**
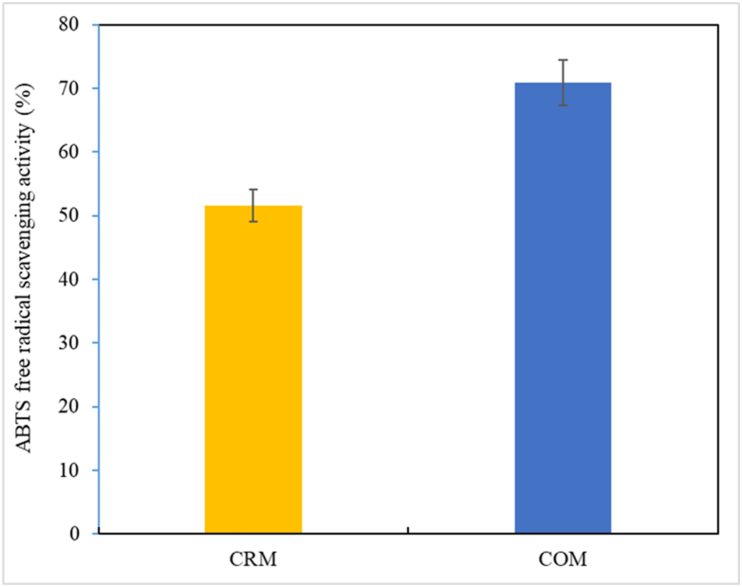
ABTS free radical scavenging activity in COM and CRM. The data indicates means with standard deviations (three samples/treatment). CRM: *Codium fragile* + Rice-based *Makgeolli;* COM: *Codium fragile* + Oat-based *Makgeolli.*^a-b^At the same temperature and pH treatment, values marked with different superscript letters are significantly different *(P < 0.05).*

The ABTS radical scavenging activity of CRM and COM are shown in Fig. 3. This activity was 51.57 % for CRM and 70.91 % for COM; that is, the activity of COM was about 1.5 times higher than that of CRM. According to reports of studies of white bread [50], and tofu [51] to which Cf as well as freeze dried Cf had been added the ABTS radical scavenging activity significantly increased as the amount of Cf increased [52]. The high antioxidant activity of Cf becomes evident from research results that show that the alcohol extract had an ABTS radical scavenging activity of 47 % depending on the extraction method [53]. *Makgeolli* undergoes fermentation, a process driven by various microorganisms such as yeast and lactic acid bacteria. These microbes play a crucial role in breaking down complex carbohydrates into simpler compounds, producing alcohol and organic acids. Some of the microorganisms involved in fermentation may have inherent antioxidant properties or produce metabolites with antioxidant effects. In a research paper on the antioxidant components and antioxidant activity of oats, Lee et al. [54] reported the existence of the active substance *avenanthramide,* a phenol component, and Kim et al. [55] reported that, among native and medicinal plant extracts, the total flavonoid content of oats was high. Ham et al. [49] reported that the ABTS radical scavenging ability of oats was 77.88–116.14 mg TEAC/100 g, and they detected polyphenol content higher than that of white rice, coix seed, and mung bean. In this study, the content of polyphenols and flavonoids, which are antioxidants, was not measured, but Kim et al. reported the detection of a polyphenol content of 117.57 mg/g and a flavonoid content of 71.60 mg/g in oat (*Avena sativa* L.). Therefore, COM *Makgeolli* is judged to contain more antioxidant components such as polyphenols and flavonoids than CRM *Makgeolli* and is also judged to have excellent DPPH/ABTS radical scavenging activity [51,55].

### 3.6 Taste tests

The results of the taste tests of CRM and COM are presented in Table 4. After 7 days of fermentation, the *Makgeolli* was stored at 4 °C for 24 h, and the color, flavor, taste, appearance, and overall preference were assessed. Although the preference regarding appearance did not differ significantly between the two groups (*P* > 0.05), CRM was preferred over COM in terms of color, flavor, taste, and overall preference (*P* < 0.05. In addition, the significant difference in the color and flavor of COM compared with those of CRM is considered to be due to the slightly darker color and savory taste resulting from the addition of oats (which has a yellowish color) (Fig. 1). According to Song et al. [37], quality changes during alcoholic fermentation of rice wine using oats, showed that the color of oat rice wine was darker brown than rice wine, which was attributed to the epidermis of oats, which was similar to the results of this study.

**Table 4:** Taste tests in CRM and COM during fermentation for 7 days. This is about the results of the hedonic test and the rating scale 1–7.

Properties	Hedonic tests				
	Color	Smell	Taste	Appearance	Overall acceptability
CRM	3.81 ± 0.75^b^	4.31 ± 0.48^b^	5.06 ± 0.25^b^	4.94 ± 0.68^a^	4.93 ± 0.68^b^
COM	5.37 ± 0.50^a^	5.88 ± 0.50^a^	6.00 ± 0.52^a^	5.50 ± 0.89^a^	6.06 ± 0.44^a^

The data indicates means with standard deviations (three samples/treatment). Within the same column, means with different letters (a-b for each fermentation time). CRM: *Codium fragile* + Rice-based *Makgeolli*; COM: *Codium fragile* + Rice/Oat-based *Makgeolli.*

## 4 Conclusions

On the final day (day 7) of fermentation in this study, both the sugar and alcohol content of COM were significantly lower (*P* < 0.05) than those of CRM. However, compared with CRM, COM contained 1.4 times more yeasts and 1.4 times more lactic acid bacteria. Regarding the antioxidant properties of DPPH and the ABTS radical scavenging ability, the antioxidant activity of COM was excellent and about 1.5 times higher than that of CRM. In addition, COM achieved a very good overall preference of 6.06 on the 7-point sensory scale. Alcohol production in CRM and COM was maintained at 10.1%–11.5 % for 4–6 days. These results suggest the possibility of shortening the fermentation process and commercial use. In other words, based on our comprehensive evaluation of COM, it can be said that it is a microbiologically excellent fermented beverage containing low levels of sugar and alcohol and a large number of yeasts and lactic acid bacteria (>6 log). At the same time, COM showed an excellent antioxidant function and sensory benefits. However, through HLPC, additional research is conducted to commercialize the product, including analysis of vitamins including various organic acids, various amino acids, and metabolites such as esters and aldehyde fusel oils generated during the fermentation process, as well as nutritional analysis, expiration date setting, and expanded sensory evaluation is required.

## CRediT authorship contribution statement

**Eun Hee Park:** Writing – review & editing, Writing – original draft, Visualization, Validation, Supervision, Software, Resources, Methodology, Investigation, Formal analysis, Data curation, Conceptualization. **Eun Bi Jeon:** Writing – review & editing. **Pantu Kumar Roy:** Writing – review & editing, Writing – original draft, Data curation. **Sung-Hee Park:** Writing – review & editing. **Shin Young Park:** Writing – review & editing, Writing – original draft, Visualization, Validation, Supervision, Project administration, Funding acquisition, Conceptualization.

## Data availability statement

Data will be made available on request.

## Ethical statement

All participants were informed that consent to participate in the study and publish their data would be assumed on completion and submission of the study questionnaire. In Korea, research directly related to health and life sciences, such as pharmacy and medicine, has traditionally required IRB (Institutional Review Board) approval. However, it has been common practice in Korea for research in the food field, including studies with sensory components, to be conducted without IRB approval, particularly when involving small groups (10 or fewer participants) and non-harmful substances. Since this was an in-house experiment conducted without receiving research funding from the government or other industries, the taste test was evaluated by researchers, so the experiment was conducted without approval from the ethics committee. Therefore, verbal informed consent was obtained from the participants before doing the experiments.

## Declaration of competing interest

None.
