# Supplementary material for: Quality characteristics and antioxidant activity of the Korean traditional rice-oat wine “Makgeolli” supplemented with green seaweed Codiumfragile
Source: Heliyon. 2024 Oct 12;10(20):e39348. doi: 10.1016/j.heliyon.2024.e39348 (PMC11620207; doi:10.1016/j.heliyon.2024.e39348)
Supplement: Multimedia component 1 [file mmc1.docx]

**Table 1.** Changes in pH and acidity (%) in CRM and COM during fermentation.

| **Fermentation time**  **(day)** | **pH** | | **Acidity (%)** | |
| --- | --- | --- | --- | --- |
|  | **CRM** | **COM** | **CRM** | **COM** |
| 0 | 5.28 ± 0.00^aA^ | 4.85 ± 0.00^aB^ | 0.20 ± 0.00^eA^ | 0.19 ± 0.00^eB^ |
| 1 | 5.08 ± 0.01^bA^ | 4.69 ± 0.00^bB^ | 0.27 ± 0.03^fA^ | 0.19 ± 0.05^eB^ |
| 2 | 3.57 ± 0.01^fB^ | 3.63 ± 0.01^efA^ | 0.86 ± 0.01^aA^ | 0.82 ± 0.02^aB^ |
| 3 | 3.54 ± 0.02^gB^ | 3.62 ± 0.01^fA^ | 0.86 ± 0.01^aA^ | 0.82 ± 0.02^aB^ |
| 4 | 3.58 ± 0.01^fB^ | 3.64 ± 0.01^eA^ | 0.80 ± 0.02^abA^ | 0.77 ± 0.01^bB^ |
| 5 | 3.62 ± 0.02^eB^ | 3.66 ± 0.00^dA^ | 0.78 ± 0.01^bcA^ | 0.71 ± 0.01^cB^ |
| 6 | 3.69 ± 0.01^cB^ | 3.67 ± 0.01^dA^ | 0.72 ± 0.00^cdA^ | 0.67 ± 0.02^dB^ |
| 7 | 3.66 ± 0.03^dB^ | 3.69 ± 0.01^cA^ | 0.68 ± 0.02^dA^ | 0.65 ± 0.01^dB^ |

The data indicates means with standard deviations (three samples/treatment). Within the same column, means with different letters (a-f for each fermentation time). Within the same row, means with different letters (A-B). The one-way ANOVA performed by t-test was carried out to evaluate the statistical significance of differences between CRM and COM samples.

CRM: *Codium fragile* + Rice-based *Makgeolli*; COM: *Codium fragile* + Rice/Oat-based *Makgeolli.*

**Table 2.** Changes in sugar content (Brix) and alcohol (%) in CRM and COM during fermentation.

| **Fermentation time**  **(day)** | **Sugar content (ºBrix)** | | **Alcohol (%)** | |
| --- | --- | --- | --- | --- |
|  | **CRM** | **COM** | **CRM** | **COM** |
| 0 | 1.6 ± 0.55^fA^ | 1.6±0.00^fA^ | 3.0 ± 0.00^fA^ | 2.5 ± 0.00^dB^ |
| 1 | 8.5 ± 0.12^eA^ | 7.2 ± 0.00^eB^ | 12.5 ± 0.00^eA^ | 11.0 ± 0.00^cB^ |
| 2 | 13.5 ± 0.06^aA^ | 8.8 ± 0.00^aB^ | 17.0 ± 0.00^aA^ | 12.0 ± 0.00^aB^ |
| 3 | 10.6 ± 0.06^bA^ | 7.7 ± 0.10^bB^ | 16.0 ± 0.00^bA^ | 12.0 ± 0.00^aB^ |
| 4 | 10.1 ± 0.12^cA^ | 7.5 ± 0.06^deB^ | 15.2 ± 0.29^cA^ | 11.5 ± 0.00^bB^ |
| 5 | 10.1 ± 0.00^cA^ | 7.5 ± 0.01^cB^ | 15.0 ± 0.00^cA^ | 11.5 ± 0.00^bB^ |
| 6 | 10.1 ± 0.00^cA^ | 7.5 ± 0.00^cB^ | 14.3 ± 0.29^dA^ | 11.5 ± 0.50^bB^ |
| 7 | 9.8 ± 0.29^dA^ | 7.4 ± 0.10^dB^ | 14.5 ± 0.00^dA^ | 11.2 ± 0.29^bcB^ |

The data indicates means with standard deviations (three samples/treatment). Within the same column, means with different letters (a-f for each fermentation time). Within the same row, means with different letters (A-B). The one-way ANOVA performed by t-test was carried out to evaluate the statistical significance of differences between CRM and COM samples. CRM: *Codium fragile* + Rice-based *Makgeolli*; COM: *Codium fragile* + Rice / Oat-based *Makgeolli.*

**Table 3.** Changes of yeast (log CFU/mL) and lactic acid bacteria (log CFU/mL) in the CRM and COM during fermentation.

| **Fermentation time**  **(day)** | **Yeast** | | **Lactic acid bacteria** | |
| --- | --- | --- | --- | --- |
|  | **CRM** | **COM** | **CRM** | **COM** |
| 0 | 4.21 ± 0.59^gB^ | 4.64 ± 0.84^cA^ | 4.37 ± 0.00^eA^ | 4.20± 1.35^fB^ |
| 1 | 4.37 ± 0.14^eB^ | 4.60 ± 0.07^cA^ | 7.32 ± 0.02^aA^ | 7.17 ± 0.03^aB^ |
| 2 | 4.44 ± 0.05^dB^ | 4.58 ± 0.06^cA^ | 6.23 ± 0.35^bB^ | 6.85 ± 0.16^bA^ |
| 3 | 4.75 ± 0.12^aB^ | 5.35 ± 0.92^bA^ | 5.14 ± 1.39^cB^ | 6.48 ± 0.01^cA^ |
| 4 | 4.54 ± 0.01^bB^ | 6.09 ± 0.71^aA^ | 4.98 ± 0.46^dB^ | 6.43 ± 0.16^dA^ |
| 5 | 4.48 ± 0.01^cB^ | 6.11 ± 0.05^aA^ | 4.93 ± 0.09^dB^ | 6.10 ± 0.02^eA^ |
| 6 | 4.48 ± 0.01^cB^ | 6.08 ± 0.07^aA^ | 4.35 ± 0.04^eB^ | 6.10 ± 0.02^eA^ |
| 7 | 4.30 ± 0.01^fB^ | 6.04 ± 0.05^aA^ | 4.28 ± 0.02^fB^ | 6.09 ± 0.18^eA^ |

The data indicates means with standard deviations (three samples/treatment). Within the same column, means with different letters (a- f for each fermentation time). Within the same row, means with different letters (A-B). The one-way ANOVA performed by t-test was carried out to evaluate the statistical significance of differences between CRM and COM samples. CRM: *Codium fragile* + Rice-based *Makgeolli*; COM: *Codium fragile* + Rice/Oat-based *Makgeolli.*

**Table 4.** Taste tests in CRM and COM during fermentation for 7 days. This is about the results of the hedonic test and the rating scale 1-7.

| **Properties** | **Hedonic tests** | | | | |
| --- | --- | --- | --- | --- | --- |
|  | **Color** | **Smell** | **Taste** | **Appearance** | **Overall acceptability** |
| CRM | 3.81 ± 0.75^b^ | 4.31 ± 0.48^b^ | 5.06 ± 0.25^b^ | 4.94 ± 0.68^a^ | 4.93 ± 0.68^b^ |
| COM | 5.37 ± 0.50^a^ | 5.88 ± 0.50^a^ | 6.00 ± 0.52^a^ | 5.50 ± 0.89^a^ | 6.06 ± 0.44^a^ |

The data indicates means with standard deviations (three samples/treatment). Within the same column, means with different letters (a-b for each fermentation time). CRM: *Codium fragile* + Rice-based *Makgeolli*; COM: *Codium fragile* + Rice/Oat-based *Makgeolli.*

**Rice (400 g) (CRM)**

**Rice (200 g) and Oat (200 g) (COM)**


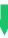


**Soaking for 12 hr and draining for 1 hr**

**Steaming for 1 hr and cooling (room temp.)**


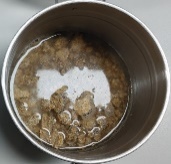


**Added *Codium fragile* (80 g)**


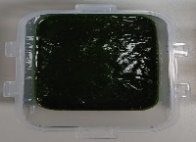


**Combination of *Nuruk* (80 g), yeast (1 g), distilled water (800 mL) Mixed**

**Fermentation at 25 ℃ for 7 days**

**Filtering and Bottling**


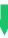

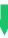

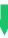

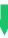

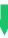

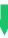

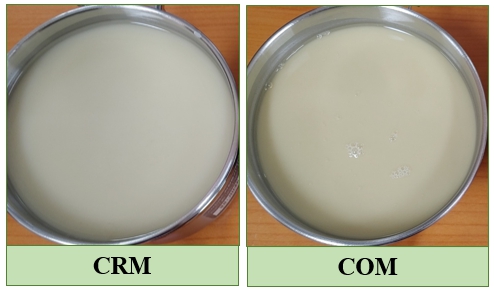

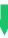

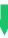

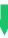

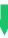


**Figure 1.** A flow diagram for the preparation of *Makgeolli* with *Codium fragile* + Oat.

CRM: *Codium fragile +* Rice-based *Makgeolli*

COM: *Codium fragile* + Oat-based *Makgeolli*


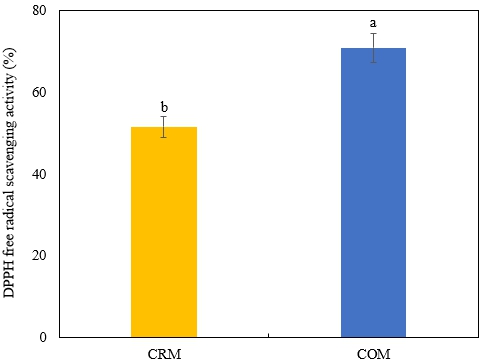


Fig. 2. DPPH free radical scavening activity in CRM and COM. The data indicates means with standard deviations (three samples/treatment). CRM: *Codium fragile* + Rice-based *Makgeolli*. COM: *Codium fragile* + Oat-based *Makgeolli.*


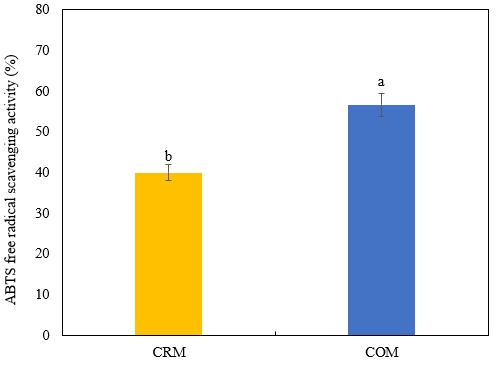


Fig. 3 ABTS free radical scavening activity in CRM and COM. The data indicates means with standard deviations (three samples/treatment). CRM: *Codium fragile* + Rice-based *Makgeolli*. COM: *Codium fragile* + Oat-based *Makgeolli.*

**<** Taste tests **>**

Please evaluate the next sensory items for the food samples according to the scale provided being

1=dislike extremely or not acceptable, 2=dislike slightly, 3=dislike moderately,

4=neither like nor dislike, the lower limit of the acceptable range, 5=like slightly,

6=like moderately, 7=like extremely

**Name : Date : . . .**

**1. Color**

|  |  | 1  dislike extremely | 2  dislike slightly | 3  dislike moderately | 4  neither like nor dislike | 5  like  slightly | 6  like moderately | 7  like extremely |
| --- | --- | --- | --- | --- | --- | --- | --- | --- |
| Control | CRM |  |  |  |  |  |  |  |
| Treat 1 | COM |  |  |  |  |  |  |  |

**2. Smell**

|  |  | 1  dislike extremely | 2  dislike slightly | 3  dislike moderately | 4  neither like nor dislike | 5  like  slightly | 6  like moderately | 7  like extremely |
| --- | --- | --- | --- | --- | --- | --- | --- | --- |
| Control | CRM |  |  |  |  |  |  |  |
| Treat 1 | COM |  |  |  |  |  |  |  |

**3. Taste**

|  |  | 1  dislike extremely | 2  dislike slightly | 3  dislike moderately | 4  neither like nor dislike | 5  like  slightly | 6  like moderately | 7  like extremely |
| --- | --- | --- | --- | --- | --- | --- | --- | --- |
| Control | CRM |  |  |  |  |  |  |  |
| Treat 1 | COM |  |  |  |  |  |  |  |

**4. Appearance**

|  |  | 1  dislike extremely | 2  dislike slightly | 3  dislike moderately | 4  neither like nor dislike | 5  like  slightly | 6  like moderately | 7  like extremely |
| --- | --- | --- | --- | --- | --- | --- | --- | --- |
| Control | CRM |  |  |  |  |  |  |  |
| Treat 1 | COM |  |  |  |  |  |  |  |

**5. Overall acceptability**

|  |  | 1  dislike extremely | 2  dislike slightly | 3  dislike moderately | 4  neither like nor dislike | 5  like  slightly | 6  like moderately | 7  like extremely |
| --- | --- | --- | --- | --- | --- | --- | --- | --- |
| Control | CRM |  |  |  |  |  |  |  |
| Treat 1 | COM |  |  |  |  |  |  |  |

**6. Comments (기타 의견 시 기술해주세요.**

Thank you very much for your participation
